# STING agonism in brain tumours: mechanisms, challenges, and therapeutic advances

**DOI:** 10.3389/fonc.2026.1679361

**Published:** 2026-04-17

**Authors:** Zeta Ioannou, Paul Cressey, Mohammed H. Ahmed, Antonios N. Pouliopoulos, Darren Hargrave, Maya Thanou

**Affiliations:** 1 School of Cancer and Pharmaceutical Sciences, King’s College London, London, United Kingdom; 2 School of Biomedical Engineering & Imaging Sciences, King’s College London, London, United Kingdom; 3 Great Ormond Street Institute of Child Health, University College London, London, United Kingdom; 4 Department of Developmental Biology & Cancer, University College London, London, United Kingdom

**Keywords:** DMG, gliomas, immunomodulation, nanoparticles, STING

## Abstract

High-grade gliomas, including diffuse midline glioma (DMG), remain some of the most aggressive and treatment-resistant brain tumours, largely due to their diffuse growth, inoperability, and profoundly immunosuppressive tumour microenvironments (TMEs). The stimulator of interferon genes (STING) pathway has emerged as a promising immunotherapeutic target, capable of activating type I interferon responses and bridging innate and adaptive immunity. This review explores the dual role of STING in tumour immunity and TME modulation, examining both canonical and non-canonical signalling pathways. We summarise advances in STING agonist development, including cyclic dinucleotides, synthetic non-cyclic dinucleotides, and metal-based compounds, and critically assess their translational potential in the context of brain tumours. While preclinical studies demonstrate robust antitumour efficacy, clinical translation remains limited by systemic toxicity, delivery constraints, and variability in STING expression across glioma subtypes. We hence offer insights into novel drug delivery approaches such as nanoparticles, liposomes, hydrogels, and focused ultrasound for overcoming the key challenges of bioavailability and blood-brain barrier penetration of the agonists. We also highlight emerging combinatorial strategies—particularly checkpoint inhibitors and epigenetic modulators—as essential to enhancing therapeutic outcomes; an outlook not previously explored for brain malignancies. Overall, we conclude that STING agonism offers a compelling strategy for immunomodulation in gliomas, but further optimisation of delivery, safety, and mechanistic understanding is crucial for successful clinical application.

## Introduction

High-grade primary gliomas affect both adult and paediatric patients with a dismal prognosis (1). Some of these gliomas are characterised by diffuse infiltrative growth patterns and tumour heterogeneity, making their therapeutic treatment difficult (2). Paediatric type diffuse high-grade gliomas, such as diffuse midline glioma (DMG) with the H3K27M mutation (WHO grade IV) are located within the brainstem. These gliomas remain inoperable and untreatable due to the highly functional area of the brain they are situated in, which prevents surgical resection (3–5). Adult-type diffuse gliomas include astrocytomas (IDH-mutant), oligodendrogliomas (IDH-mutant and 1p-19q codeletion), and glioblastomas (GBM, IDH-wildtype) (6). The current treatment strategies for the adult-type DMG remain ineffective post-resection, due to the tumours’ genetic heterogeneity and their CNS invasiveness; largely relying on radiotherapy and chemotherapy such as temozolomide (TMZ) for treatment (7).

Immunotherapy breakthroughs in the treatment of solid tumours, aim to boost the immune responses to eliminate cancer cells in infiltrated tissues and treatments, directly targeting the tumour microenvironment (TME) (8, 9). The use of monoclonal antibodies has changed the therapeutic landscape of solid tumour therapy. In the context of brain tumours, immune checkpoint inhibitors targeting programmed cell death protein-1 (PD-1; nivolumab), and cytotoxic T-lymphocyte associated protein 4 (CTLA-4; ipilimumab) have been explored in clinical trials though with limited success for high grade gliomas (10–12). A phase III randomised open-label trial of 369 patients compared the effects of nivolumab to the anti-angiogenic monoclonal antibody bevacizumab in patients presenting with a first occurrence of glioblastoma (NCT02017717), but failed to demonstrate any clinical benefit, with no improvements in overall survival benefit (13). The limited efficacy of these monoclonal antibodies is likely due to multiple factors including marked genetic and antigenic heterogeneity, as well as the paucity of infiltrating T cells to these tumours due to the presence of the blood-brain-barrier (13). This highlights that the immunosuppressive ‘cold’ TME of high-grade gliomas may limit responses to these therapeutic agents alone (14, 15). This has given rise to other immunotherapies to boost immunosurveillance, namely the stimulator of interferon genes (STING) pathway and its potential agonists.

STING agents increasing the activity of the pathway, are thought to elicit robust antitumor immune responses by activating innate immunity, bridging to adaptive immune mechanisms, and hence boosting immunosurveillance within such TMEs (16–19). However, the clinical translation of STING agonists in brain tumours remains limited, mainly due to the need for direct intratumoural administration and the lack of mechanistic understanding of STING activation within the CNS (20). This review provides a comprehensive overview of the mechanism of the STING pathway within primary brain tumours, current strategies for STING agonist delivery to the brain, along with clinical advancements made in the field and the challenges that remain in translating these approaches to the clinic. We also offer a new outlook on combinatorial therapies and nanocarriers for overcoming limitations of the agonists in terms of delivery, including strategies to overcome the blood-brain-barrier.

## Definition and role of STING

**Figure 1:**
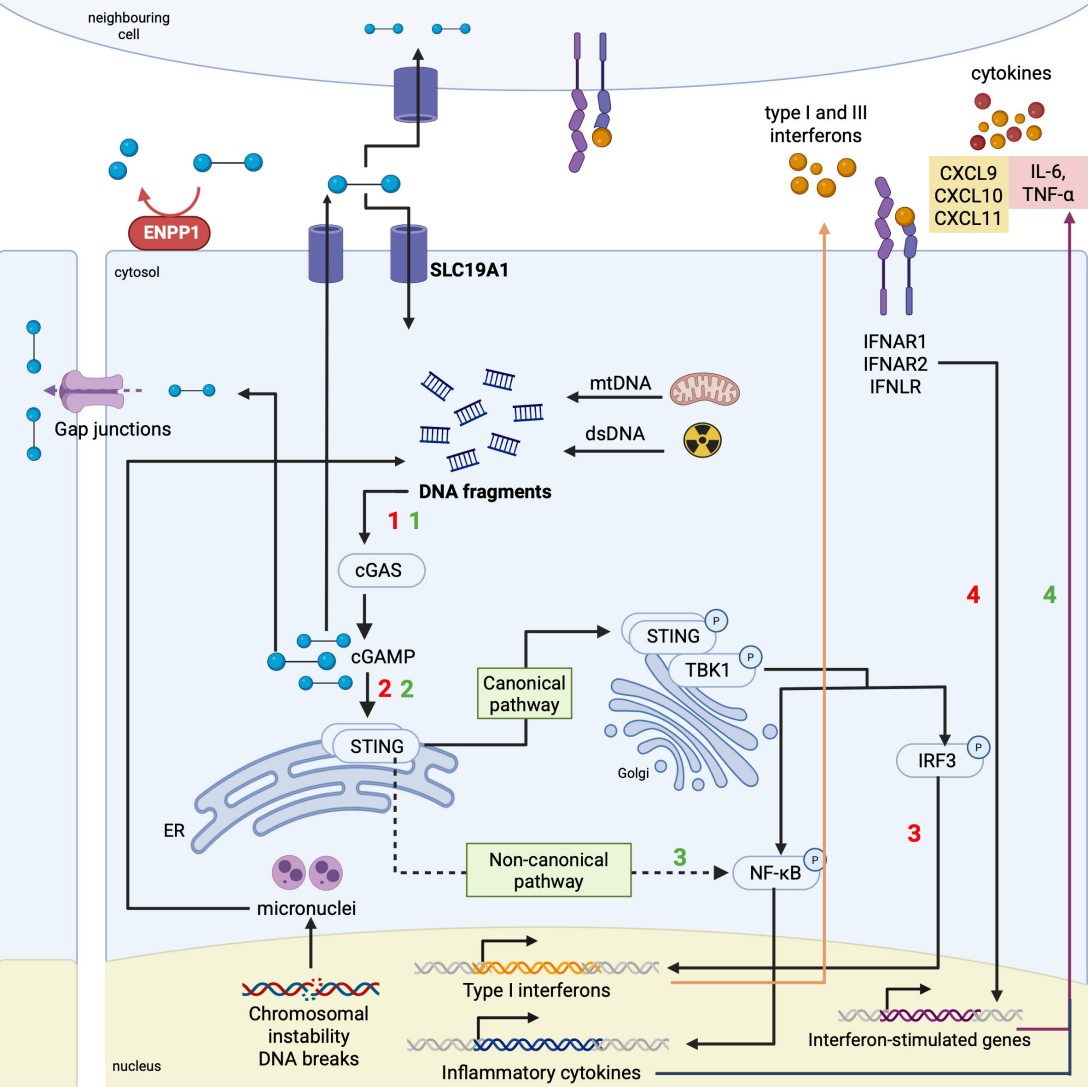
The STING activation and downstream cascade. The canonical and non-canonical arms of the STING pathway are both initiated via the detection of double-stranded DNA (ds-DNA) by cyclic GMP-AMP synthase (cGAS). In highly stressful cellular conditions such as in the case of cancer, free ds-DNA in the cytosol can arise from mitochondrial instability (mtDNA), DNA damage from radiotherapy or chemotherapy that the patient is receiving and chromosomal instability from the cancer cells themselves. cGAS detects these DNA fragments to release natural STING agonist, cyclic 2’3- GAMP-AMP (cGAMP). cGAMP can also be transported to neighbouring cells via gap junctions and active channels with the main transporter of interest therapeutically being SLC19A1. This facilitates the activation of the cascade in adjacent cells which may not contain free cytosolic DNA. Upon generation, cGAMP subsequently binds to binding pockets on the STING protein within the endoplasmic reticulum (ER), contributing to its translocation to the Golgi apparatus and subsequent phosphorylation by TANK-binding kinase 1 (TBK1) in the canonical pathway. This phosphorylated STING (p-STING) mediates the recruitment and phosphorylation of transcription factor interferon regulatory factor 3 (IRF3). This mediates the translocation of IRF3 to the nucleus to transcribe *IFNB1*, resulting in the release of type I IFNs. IFNs will bind to their cognate receptors, the IFN receptors (IFNAR), inducing downstream signalling cascades. Among others, these signals contribute to the transcription of interferon-stimulated genes to exert their anti-tumourigenic effects via the release of pro-inflammatory cytokines for downstream effector T cell recruitment. In the non-canonical pathway, NF-κB activation predominates and stimulates the downstream cascade. For negative feedback regulation of the cascade, phosphodiesterase enzyme ENPP1 is responsible for the degradation of cGAMP, restricting its extracellular half-life. Figure created in Biorender.com.

The canonical cGAS/STING pathway (cyclic GMP-AMP synthase/STING pathway), has been identified as one of the key signalling components of cytosolic DNA sensing (21, 22). It is initiated following sensing of double-stranded DNA by DNA sensor cGAS (**Figure 1**, step 1). Following a series of complex downstream catalytic steps (further expanded in **Figure 1**, legend), STING becomes activated by mostly cyclic dinucleotides (CDNs), including its innate ligand cyclic GAMP-AMP (cGAMP), generated by cGAS (**Figure 1**, step 2) (16, 23). The downstream transcriptional steps result in the release of type I interferons (**Figure 1**, step 3, red). Upon binding to the IFN receptor on myeloid cells such as macrophages, type I interferons induce the expression of interferon stimulated genes (**Figure 1**, step 4) (24, 25).

The noncanonical STING pathway of activation is independent of cGAS-mediated cGAMP generation, and is induced by DNA damage where NF-κB signalling dominates. STING binds and stimulates IκB kinase to trigger the transcriptional activation of NF-κB (**Figure 1**, step 3, green), contributing to the transcription and translation of pro-inflammatory cytokines such as interleukin-1β and tumour necrosis factor alpha (TNF-α) (**Figure 1**, step 4, green) (23, 24). This DNA damage can be induced by chemotherapeutic agents such as etoposide and temozolomide (TMZ), used in the treatment of high-grade gliomas (26, 27).

Activation of both STING pathways can also lead to the production of other pro-inflammatory cytokines, including CXCL10 and CXCL9 (28, 29). CXCL10 and CXCL9 are interferon-inducible chemokines that bind to receptors expressed on activated T cells and natural killer (NK) cells, such as CXCR3 (30, 31). The CXCL10/CXCR3 and CXCL9/CXCR3 axes are one of the end results of the STING pathway, which promote the recruitment and trafficking of cytotoxic effector T cells and NK cells into the TME. This boosts immunosurveillance within the TME, encouraging cytotoxicity by the anti-tumour immune response (31, 32).

## Role of STING in the anti-tumour response: immune activation and tumour microenvironment modulation

The STING pathway plays a pivotal role in coordinating anti-tumour immunity, particularly within the immunosuppressive TME of high-grade gliomas. Effective immune-mediated tumour control is mediated by CD8^+^ cytotoxic T lymphocytes, NK cells, M1-polarised macrophages, and antigen-presenting cells (APCs), which exert anti-tumour effects through cytokine secretion and cytotoxicity (33–35). In contrast, regulatory T cells (Tregs), M2 macrophages, and myeloid-derived suppressor cells (MDSCs) facilitate tumour progression by dampening effector responses and promoting angiogenesis via cytokines such as interleukin-10 and TGF-β (36, 37).

STING activation induces type I interferon (IFN-I) production, primarily in myeloid-lineage cells, such as microglia and dendritic cells (DCs) (38). STING1 is highly expressed in immune and stromal cells of the TME (39, 40) and its activation in DCs has been shown to initiate innate immune responses essential for tumour control (41). Tumour-resident CD8α^+^ DCs are major IFN-I producers following STING activation, promoting cytotoxic T cell recruitment and activation (16, 42, 43). DNA and CDNs released from dying tumour cells can also trigger STING activation in nearby APCs via the transporter SLC19A1, contributing to a chain of STING activation in the TME (16, 39, 44).

**Figure 2:**
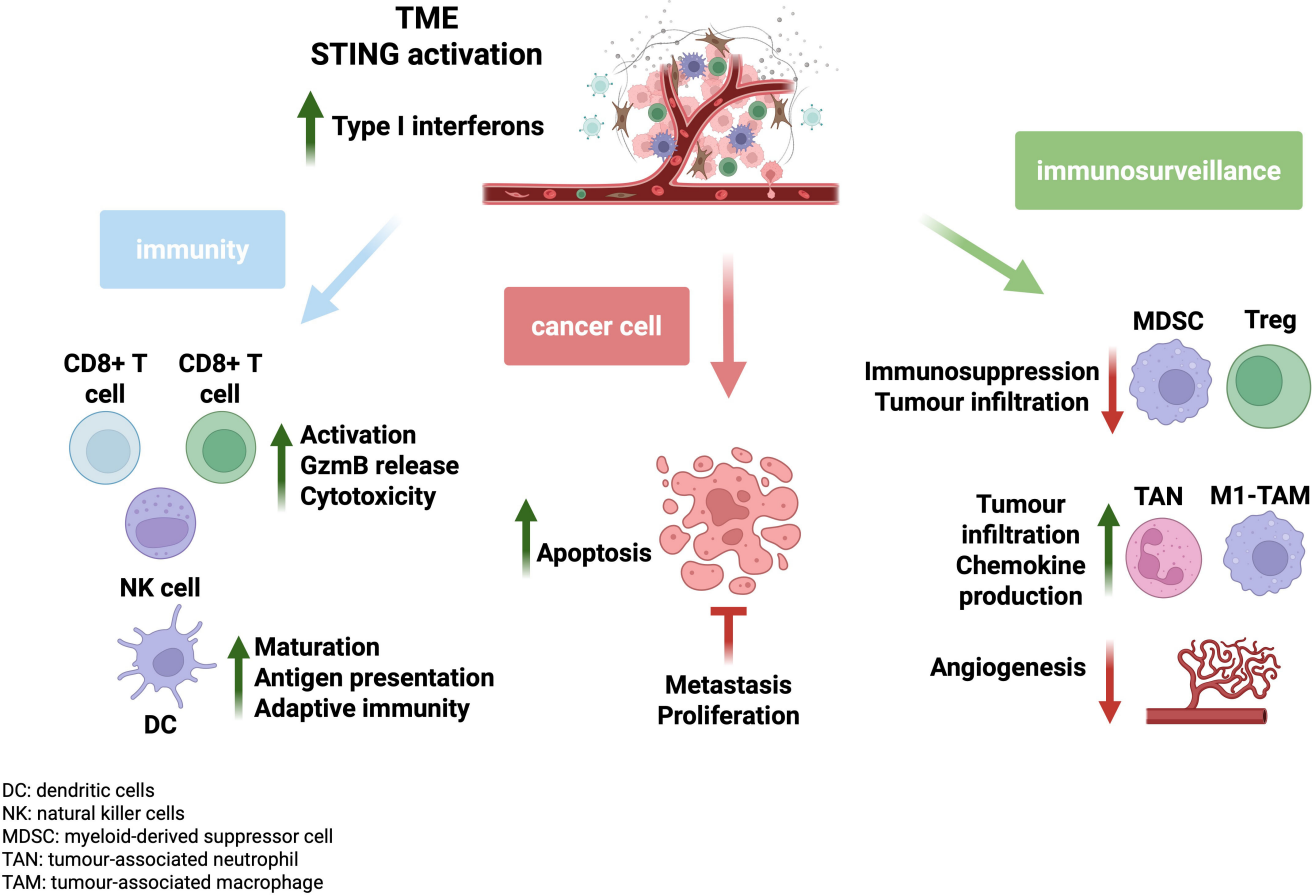
Modifications of the tumour microenvironment with increasing type I interferon activity. An increase in type I interferons (IFNs) within the tumour microenvironment (TME) of solid tumours can contribute to anti-tumourigenic activity through various cellular moieties. Type I IFNs can result in the activation of cytotoxic T cells (CD8^+^) and Natural Killer (NK) cells, and T helper cells (CD4^+^). Cytotoxic cells can use their granule exocytosis pathway to target cancer cell death, operating through the delivery of cytotoxic granules to the surface of the cancer cells such as perforin. Perforin facilitates the delivery of granzyme B to the cancer cell cytosol via permeating through the cancer cell surface, promoting cell apoptosis either through BID-dependent mitochondrial permeabilisation, or through direct caspase 3 cleavage and subsequent activation of the caspase apoptotic cascade. Type I IFNs can also induce the maturation of antigen presenting cells such as dendritic cells (DCs), increasing their ability to cross-present tumour-specific antigens to T helper cells for cytotoxic cell activation and ultimately tumour cell clearance. STING deficiency in DCs results in failed DC responses to prime T cells. Type I IFNs can also reduce the population of regulatory T (Treg) cells within the TME, as Tregs can suppress antitumour immunity and hinder immunosurveillance further in ‘cold’ tumours. They can also inhibit the proliferation of myeloid-derived suppressor cells (MDSCs) as the latter can promote angiogenesis while fostering metastasis. These effects occur while type I IFNs upregulate antitumour cell populations such as tumour-associated neutrophils (TANs) and tumour-associated macrophages (TAMs) and facilitating their polarisation towards the anti-tumourigenic N1 and M1 phenotypes respectively. They in turn can become activated and secrete a plethora of pro-inflammatory cytokines for T cell activation and phagocytic/cytotoxic activity. Figure created in Biorender.com.

STING agonism reprogrammes M2 macrophages toward an M1 phenotype, enhancing TNF-α and interleukin secretion and enabling CD8^+^ T cell priming (38, 44). Additionally, tumour-derived 2′3′-cGAMP promotes T cell transcription factor expression in NK cells and upregulates IL-15 and its receptor, boosting NK cell cytotoxicity (45–47). IFN-I further attenuates immunosuppressive populations by suppressing Tregs and MDSCs (48), while CD8^+^ T cells, supported by CD4^+^ T cells, engage STAT3–Granzyme B signalling to eliminate tumour cells. A higher CD8^+^/CD4^+^ ratio correlates with improved prognosis in glioblastoma, whereas elevated M2 macrophage infiltration is associated with poorer outcomes (38, 48, 49). STING agonists can reprogramme suppressive myeloid cells more effectively than depletion-based approaches (**Figure 2**) (13, 48, 50).

Despite STING’s immunomodulatory benefits, its expression in glioma cells is often minimal, possibly due to mutations in STING1 or cGAS, or epigenetic silencing (39, 40). Demethylating agents such as Zebularine have been shown to restore STING expression in glioblastoma lines (20, 51). *In vivo*, the 2’3’-cGAMP-based STING agonist 8803, was found to increase median survival in QPP8, an immune checkpoint blockade-resistant model of glioblastoma multiforme, where 100% of glioblastoma-bearing mice were rid of the tumours (17). This was accompanied by modifications in the glioblastoma TME, notably a 4-fold increase in tumour-infiltrating CD8+ T cells and NK cells (40).

Beyond immune modulation, STING-induced IFN-I can downregulate BCL-2, upregulate Bax, and induce apoptosis in the tumour microenvironment through activation of the STING pathway in Th1, Th2 and Th17 T cells (52). This was demonstrated through treatment of primary T cells for 12hrs with small molecule STING agonist 10-carboxymethyl-9-acridanone (CMA), resulting in the triggering of caspases 3/7; both hallmarks of apoptotic cell death (52). STING pathway-induced IFN I release, was also found to suppress tumour angiogenesis and metastatic spread in different *in vivo* models of multiple myeloma, in a lung carcinoma allograft model and a 3’-methylcholanthrene (MCA)-induced sarcoma model. Loss of type I IFN signalling pathways was shown to accelerate tumour initiation and progression, whereby the total number of splenic NK cells following treatment with IL-2 was reduced in IFNAR2 deficient mice from 2.47±0.46 to 3.7±0.81 x 10^6^ cells per spleen (53). Thus demonstrating that NK generation and proliferation was largely IFN-I dependent (16, 48).

## STING pathway activation in high-grade gliomas

Current clinical therapy for high-grade gliomas remains palliative, with chemotherapeutic agents etoposide and TMZ frequently used in these regimens (26). Due to their respective mechanisms of action, double-stranded breaks within the tumour DNA are induced. This in turn results in cancer cells undergoing necrosis and/or apoptosis if no further repair is available, resulting in the release of extracellular DNA fragments (54, 55). In addition to this, the genomic instability of cancer cells can result in stalled replication forks, which in turn result in DNA structure-specific endonuclease MUS81 cleaving dysfunctional DNA structures. This amplifies the accumulation of DNA in the cytoplasm, in turn triggering the non-canonical STING cascade (23). High-grade glioma cancer cells are also proposed to experience metabolic stress whereby nuclear and mitochondrial-DNA is released in the cytoplasm (56). It has previously been shown that mitochondrial-DNA can activate the cGAS/STING pathway in its own right (57).

Additional means of extracellular DNA release involve the release of micronuclei due to cancer cell chromosomal disaggregation. This in turn triggers dsDNA sensing and the downstream STING pathway (24). Indeed, high chromosomal instability has been observed in DMG, highlighting the potential of the STING pathway as a therapeutic avenue for this high-grade glioma type (24). DMG tumours also possess a mutated form of p53, a central component of the DNA damage response, in turn interacting directly with TBK1 and disabling the formation of the STING-TBK1-IRF3 complex, hindering the formation of type I interferons in this tumour (24). This facilitates the deficits observed in immunosurveillance.

Diffuse gliomas vary in their genetic and epigenetic profiles, which in turn is reflected in their differential expression of the cGAS/STING pathway (58). For example, glioblastoma multiforme (GBM) was found to not only express the cGAS/STING pathway, but also that treatment with STING agonists to orthotopic GBM tumours, resulted in an increase in survival in GL-261 and CT-2A orthotopic syngeneic *in vivo* models, implying expression of the pathway and responsiveness in the GBM TME (40). In H3K27M diffuse midline gliomas, STING pathway expression is less widely characterised, with the consensus being epigenetic suppression of the pathway. Particularly, it is hypothesised that cg16983159 methylation in glioma cells can lead to the silencing observed. Myeloid, endothelial and stromal cells in the TME however were found to still express STING (39). More recently, Zhong and colleagues found that expression of STING is significantly higher in high-grade gliomas, and the relapse scores in patients based on the transcriptional and translational expression levels of the STING protein specifically predict poorer survival clinical outcomes (58).

## STING agonism as a therapeutic approach in brain malignancies

Immunotherapies have recently gained interest for high-grade glioma treatment, albeit with little clinical benefit. This comes as an attempt to transition from traditional chemotherapeutic approaches, alleviating the adverse effect burden for patients. The CheckMate 908 trial (NCT03130959; n=166, five-arm open-label completed in the United States) investigated the efficacy of the combination therapy of immune checkpoint inhibitors, with fully humanised IgG monoclonal antibody against PD-1 nivolumab, and that of anti-CTLA4 IgG ipilimumab. However, it found no improvement in patient survival rate with newly diagnosed DMG or with recurrent or progressive glioblastoma when compared to standard chemotherapy (15). The limited effectiveness of this approach was explained to be partly due to the immunologically suppressive nature of their TME, whereby spontaneous anti-tumour T cell responses are sparse (20). This sparked a research interest in developing novel immunotherapies and based on the current knowledge regarding the STING pathways, STING agonists were developed. These molecules have been found to induce the expression of PD-L1 on the tumour cell surface. It has been suggested that STING-targeted strategies in treating high-grade glioma cells irrespective of STING protein levels and STING pathway activation are a promising avenue for therapy (58). This provided a rationale for the synergistic use of STING agonists with immune checkpoint inhibitors, with recent research directed at the synthesis of different classes of STING pathway agonists (59, 60).

## Cyclic dinucleotides and small molecules STING agonists

In the context of brain malignancies, the intracranial injection of CDN agonist c-di-GMP into a murine orthotopic GL261-luc-glioma model was found to improve the survival rate of the animals, inhibiting glioma growth by 20% while improving the migration of T cells to the tumour region (16). Namely, the STING agonist increased the proportion of CD4^+^, CD8+ and CD11c^+^ brain-infiltrating leukocytes compared to untreated controls up to three-fold (16, 23).

2’3’-cGAMP, being produced by mammalian cGAS intracellularly, shows high affinity for all hSTING isoforms (61). Despite natural CDNs being the natural agonists for STING, their translatability as clinical drug candidates is poor due to their susceptibility to hydrolytic cleavage by phosphodiesterase enzymes (PDEs) (62). The focus on CDN research in the STING pathway is currently on synthesising non-hydrolysable CDN analogues, the efficacy of which has already been demonstrated (63).

**Figure 3:**
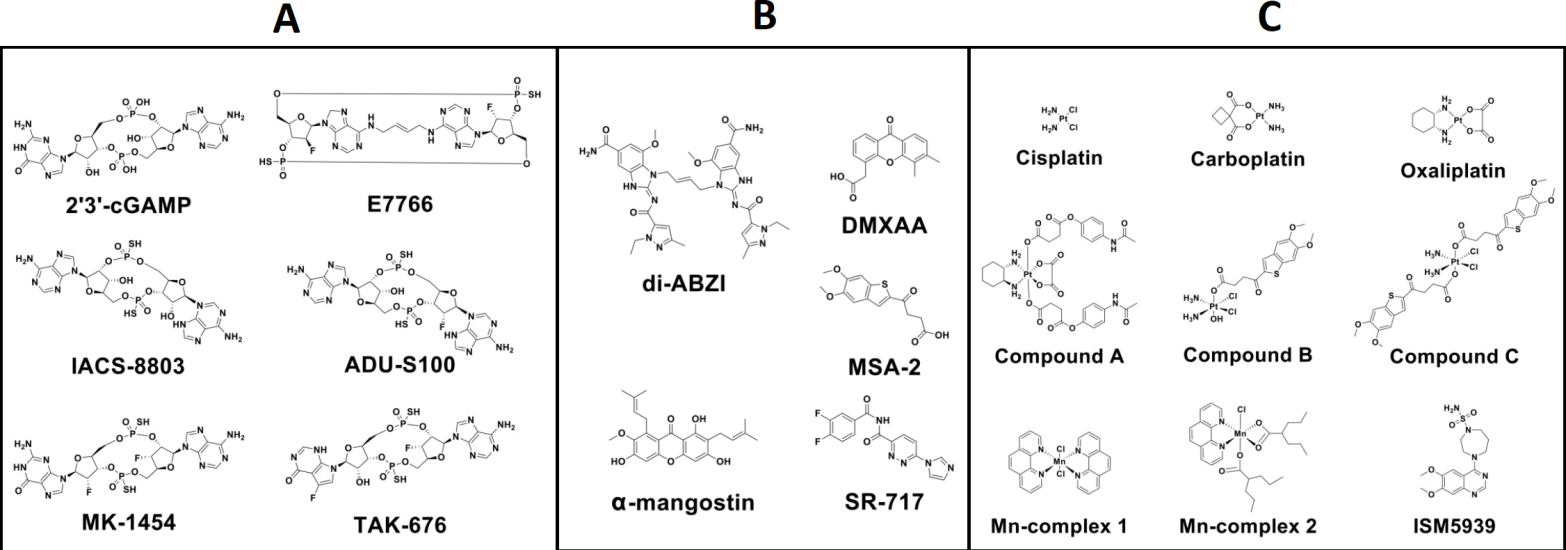
Chemical structures of drugs interacting with the cGAS/STING pathway. The various agonists to the cGAS/STING pathway investigated in the context of solid cancers with **(A)** cyclic dinucleotide (CDN)-based agonists as analogues of the natural STING agonist 2’3’-cGAMP; **(B)** Non-CDNs, small molecule-based STING agonists which are resistant to cleavage by phosphodiesterase enzyme ENPP1; and **(C)** metal-based STING agonists which are thought to interact synergistically with widely available metal-based chemotherapeutic agents such as the platins to augment therapeutic effects. Figure created in Chemdraw.

CDN analogues containing 2’5’, 2’3’ and 3’5’ phosphorothioate bonds mimic the structural features of natural CDNs whilst resisting degradation by PDEs, namely ectonucleotide pyrophosphatase phosphodiesterase 1 (ENPP1) (64). The mixed phosphorothioate bonds improve the stability and lipophilicity of the molecules while displaying high affinity for hSTING (18). Chemically, the substitution of the nonbridging oxygen atoms at the phosphate bridge with sulphur atoms enhances resistance to degradation by ENPP1, enhancing cellular uptake and the delivery of the drug in the cytosol (64–66). The structures of some CDN agonists being studied in pre-clinical and clinical models are shown in **Figure 3**.

The drug IACS-8803 (**Figure 3**) in this class has been shown to induce complete tumour regression in canines with spontaneously arising glioblastoma when administered intracranially (44, 67). In fact, IACS-8803 showed superior regression of the contralateral tumour versus the innate STING agonist 2’3’-cGAMP. In the canine translational clinical trial, there was over 75% tumour reduction at 20mg doses, and a complete response after a second 20mg dose. Median progression-free survival was 14 weeks (44).

MK-1454 (**Figure 3**) developed by Merck, also known as ulevostinag® has been investigated for feasibility trials in advanced and metastatic solid tumours. Intratumoural ulevostinag has completed phase I clinical trials in combination with pembrolizumab (NCT03010176, n=156, six-arm). When ulevostinag^®^ was administered systemically, it displayed poor pharmacokinetics and degradation (41, 68). This may be the key reason for the poor performance of systemically administered CDNs. In addition to that, systemic administration may lead to off target STING activation and inflammation, highlighting the need of smart delivery systems.

The combination of pembrolizumab and MK-1454 (**Figure 3**) in a phase 1 trial demonstrated only a partial response of 24% overall response rates (69). MK-1454 monotherapy did not demonstrate any response. The result of this trial is the only evidence of CDN synergistic effect in solid tumours.

TAK-676 (**Figure 3**) is a novel synthetic phosphorothioate STING agonist designed for stability for intravenous administration, developed by Takeda pharmaceuticals (41). TAK-676 is currently in phase I/II clinical trials (NCT04420884, n=248, eight-arm) for the treatment of metastatic and advanced solid tumours, including brain. A detailed breakdown of the various past and present clinical trials involving CDN STING agonists for solid tumours has been shown in **Table 1**. Until now, no CDN has been tested in Phase II or III clinical trials. It is yet to be found if synthetic phosphorothioate CDNs can overcome the issues with biological stability and bioavailability, and show a measurable tumour response, or whether an advanced drug delivery system can improve tumour bioavailability and response.

**Table 1:** A summary of the various clinical trials involving STING agonism in solid tumours including brain.

Trial number	Trial	Design	Condition	Mechanism of action	Mode of administration	(Industrial) partner	Phase
NCT05549804	A Study of Intratumoural KL340399 in Patients with Advanced Solid Tumours	Single-centre, open-label, dose-escalation n=30	Advanced solid tumours	Non-cyclic dinucleotide STING agonist	intratumoural	Sichuan Kelun Pharmaceutical Research Institute Co., Ltd.	1 (active)
NCT04144140	Study of Intratumorally Administered Stimulator of Interferon Genes (STING) Agonist E7766 in Participants With Advanced Solid Tumours or Lymphomas – INSTAL-101	Multi-centre, open-label, dose-escalation n=24	Advanced solid tumours	Macrocycle- bridged STING agonist	intratumoural	Eisai Inc.	1/1b (terminated due to business directions)
NCT05978492	A Study of TXN10128 in Subjects With Solid Tumours	Multi-centre, open-label, dose-escalation and expansion n=96	Locally advanced (unresectable) or metastatic solid tumours	ENPP1 inhibitor	oral	Txinno Bioscience Inc.	1 (active)
NCT06724042	Study of ISM5939 in Patients With Advanced and/​or Metastatic Solid Tumors	Multi-centre, open-label, dose escalation and expansion N=159	Advanced and/or metastatic solid tumours	ENPP1 inhibitor	oral	InSilico Medicine Hong Kong Limited	1 (active)
NCT02675439	Safety and Efficacy of MIW815 (ADU-S100) +/​- Ipilimumab in Patients With Advanced/​Metastatic Solid Tumours or Lymphomas	Multi-centre, open-label, safety and efficacy n=47	Advanced/metastatic solid tumours	Cyclic dinucleotide analogue STING agonist	intratumoural	Chinook Therapeutics, Inc. (formerly Aduro)	1 (terminated)
NCT04609579	Study of SNX281 in Subjects With Advanced Solid Tumours and Lymphoma	Open-label, first-in-human n=27	Advanced solid tumours	Small molecule STING agonist	intravenous	Stingthera, Inc.	1 (terminated due to sponsor decision)
NCT04420884	A Study of Dazostinag as Single Agent and Dazostinag in Combination With Pembrolizumab in Adults With Advanced or Metastatic Solid Tumours (iintune-1)	Multi-centre open-label, safety and tolerability n=248	Advanced/metastatic solid tumours	Small molecule STING agonist	intravenous	Takeda	1/2 (active)
NCT03172936	Study of the Safety and Efficacy of MIW815 With PDR001 in Patients With Advanced/​Metastatic Solid Tumours or Lymphomas	Multi-centre, open-label, safety and efficacy n=106	Advanced/ Metastatic solid tumours	Cyclic dinucleotide analogue STING agonist	intratumoural	Novartis Pharmaceuticals	1b (terminated due to sponsor decision)
NCT03010176	Study of Ulevostinag (MK-1454) Alone or in Combination With Pembrolizumab (MK-3475) in Participants With Advanced/​Metastatic Solid Tumours or Lymphomas (MK-1454-001)	Multi-centre, open-label, safety n=156	Advanced/ metastatic solid tumours	Cyclic dinucleotide analogue STING agonist	intratumoural	Merck	1
NCT03843359	A First Time in Human (FTIH) Study of GSK3745417 Administered to Participants With Advanced Solid Tumours	Open-label, first-in-human, n=97	Advanced solid tumours	Small molecule STING agonist	intravenous	GSK	1 (active)
NCT05377996	A Study of XMT-2056 in Advanced/Recurrent Solid Tumours Expressing HER2	Multi-centre, first-in-human, dose escalation and expansion n=319	HER-2 positive solid tumours	Non-cyclic dinucleotide STING agonist antibody-drug-conjugate	intravenous	Mersana Therapeutics	1 (active)

The first small molecule STING agonist to enter clinical trials in 2013 was non-CDN, 5,6-dimethylx-antheonone-4-acetic acid (DMXAA, vadimezan). However, it demonstrated poor efficacy against solid tumours either when administered alone or with conventional chemotherapy in the phase III ATRACT-1 trial (NCT00662597, n=1285, two-arm). The median overall survival in the treatment group was merely 13.4 months versus 12.7 months in the placebo arm. Though it is not explicitly stated what tumours the treatments were tested on, their potential for brain malignancies has been highlighted elsewhere with *in vivo* data available (70). Specifically, intraperitoneally administered DMXAA (25mg/kg) cured 50% of subcutaneous GL261 tumours in mice (71). The same dose in intracranial GL261 tumours however displayed negligible effects (71). The limited benefit is attributed to the poor permeation of DMXAA through the blood-brain-barrier *in vivo*, and the poor ability of the drug to bind to hSTING compared to murine STING (20).

Benzothiophene oxobutanoic acid (MSA-2, **Figure 3B**) was also found to selectively bind and activate STING through its ability to phosphorylate both TBK1 and IRF3 downstream in the cascade. Dimerised MSA-2 interacts through their aromatic cores before binding as a complex to STING at the same site as 2’3’-cGAMP. This mode of binding also stabilises a close-lid conformation such as that induced by the innate STING agonist, 2’3’-cGAMP, indicating activation of the STING dimer (46, 72). MSA-2 was found to increase the production of IFN-β and proinflammatory cytokines at the tumour site consistent of STING pathway activation. The ability of this non-CDN STING agonist to induce its anti-tumoural effects through oral administration demonstrates the clinical translatability of these agents for brain cancers conditional to the bypassing the intratumoural route of administration (62). More recently, γ-butyrolactone derivatives of MSA-2 have been investigated for their potential to act as oral prodrugs whereby they can form inclusion complexes with cyclodextrin nanoparticles and target tumour myeloid cells. They in turn are thought to demonstrate improved pharmacokinetic and solubility properties (73).

There appears to be a recurrent failure in clinical applications due to inconsistent efficacy in the modulation of the TME. It is likely that the systemic delivery of these agents limits their bioavailability within the tumour itself to carry out their function. This is a consideration that needs to be addressed for all STING agonists systemically, as it might be that the modest therapeutic benefit seen with non-intratumoural approaches are in part, due to this.

## Indirect STING activation

As previously mentioned, ENPP1 is the main phosphodiesterase enzyme responsible for the degradation of CDNs in the circulation, acting as a negative regulator of the pathway and limiting the systemic delivery of CDN STING agonists. The co-administration of ENPP1 inhibitors alongside 2’3’-cGAMP has been suggested as a strategy for indirect STING pathway activation while improving the pharmacokinetics of CDN STING agonists (74). Monotherapy with orally administered ENPP1 inhibitor ISM5939 (30mg/kg), displayed a 67% tumour growth inhibition in an *in vivo* model of adenocarcinoma (75). This illustrated the possibility that dsDNA from the leaky tumour is sufficient for the activation of the downstream STING pathway, if the natural intrinsic CDN agonists of STING are protected against degradation (75). ISM5939 and another ENPP1 inhibitor SR-8541A, are currently undergoing phase I clinical trials for local advanced or metastatic solid tumours in an orally available formulation (NCT06063681, n=25, single-arm) (**Table 1**). High-grade glioma-specific data for this therapy is not yet available and is unclear whether it has been included in the clinical trial currently underway.

Otherwise, β-arrestin 2 is widely characterised as a molecule interacting heavily with G-protein-coupled receptors in their downstream signalling cascades. Recent evidence suggests that β-arrestin 2 can interact with cGAS to promote the more efficient binding of dsDNA and enhance the production of cGAMP as a result (76). This interaction modifies β-arrestin 2 through deacetylation at Lys171, enhancing the activation of the cGAS/STING pathway (p<0.003) versus control, and promoting the generation of IFN-β. Nonetheless, this pathway has not yet been leveraged in cancer therapeutic applications, highlighting the need for further research in this area (76). A new study identified stromal interaction molecule 1 (STIM1) as the Ca^2+^ sensor that anchors STING to the endoplasmic reticulum. STIM1-deficient mice were found to spontaneously activate the STING pathway, accompanied by type I interferon generation (74, 77).

These STING agonists, provide insight into the development of cGAS/STING pathway agonists as combination therapies that would be relevant in the clinic through a multitude of different interactions with the cascade.

## Metal-based agents involvement in STING activation

A new research direction to indirectly activate the cGAS/STING pathway has emerged, demonstrating the promise of metal complexes as agents which can indirectly activate the cGAS/STING pathway. Platinum-based chemotherapeutic agents are alkylating-like drugs that cross-link the tumour DNA, interfering with the DNA repairing mechanism and in turn inducing DNA damage and ultimately cancer cell apoptosis (78). The resulting tumour DNA fragments being released can then activate the cGAS/STING pathway through dsDNA detection by cGAS.

These agents have been implicated in the modulation of anti-tumour immunity and tumour invasiveness through the downregulation of matrix metalloproteinase (MMP) enzymatic activity, as well as anti-angiogenic effects (79). The gold standard of platinum-based medicines for brain tumours, namely carboplatin, cisplatin, and oxaliplatin have been found to exhibit clinical benefit (80–83). Indeed, it has been reported that cisplatin for the treatment of high-grade gliomas, namely glioblastoma, could exert its side effects via the initiation of the cGAS/STING pathway through the upregulation of cGAS and STING expression in antigen-presenting cells (84, 85). The structure of some metal-based complexes that can interact with/activate the STING pathway are shown in **Figure 3C**.

Novel platinum drugs have utilised platinum (IV) over conventional platinum (II) due to greater overall stability in the circulation and bioreductive activation. This in turn allows for a greater proportion of the drug to arrive at the target site intact (86, 87). A novel platinum (IV) complex, named compound A in **Figure 3C**, composed of oxaliplatin and acetaminophen, was found to induce STING activation and mtDNA damage, resulting in mitochondrial membrane remodelling, ultimately resulting in cancer cell pyroptosis *in vivo* and *in vitro* (88). Two additional platinum (IV) complexes, compounds B and C are composed of STING agonist MSA-2, and when administered intravenously in an *in vivo* model of pancreatic adenocarcinoma, were found to increase the frequency of NK cells by 10% versus control, and increase CD4^+^ and CD8^+^ T cell populations by 5% and 10% respectively (89). This highlights platinum-based complexes as agents interacting with the STING cascade, illustrating the combinatorial benefit of their drug-induced DNA damage with STING agonism.

Other metal complexes were also found to interact with the cGAS/STING pathway. Particularly, a close dependency between manganese (Mn^+2^) and the cascade has been uncovered (90). Mn^+2^ has a variety of effects on the pathway: the activation of cGAS directly resulting in the generation of CDN 2’3’-cGAMP non-canonically and in a non-dsDNA-related manner; and sterically enhancing the binding of ability of the STING dimer to cGAMP for its downstream activation, adopting a more favourable conformation for binding (91). All of which were found to enhance the generation of type I IFNs, contributing to the anti-tumoural function of immune cells mentioned in earlier sections of this review (92). *In vivo* data demonstrated that in a murine orthotopic hepatocarcinoma model, intravenously administered manganese chloride, the number of tumour nodules in the liver reduced by 50% versus control, with an over 10% increase in IFN-γ^+^CD8^+^ T cells (93). The resulting type I interferons were also found to downregulate mitochondrial dihydroxyacid dehydrase activity in the tumour cells and induce lipid peroxidation, thus increasing reactive oxygen species levels and inducing ferroptosis of the malignant cells (94).

Cai and others synthesised Mn^+2^ complexes (**Figure 3C**) with 1,10-phenanthroline and valproic acid, where the former is a DNA intercalator and the latter a histone deacetylase (HDAC) inhibitor. They hence not only amplified the activation of the cGAS/STING pathway but also induced DNA damage while inhibiting DNA repair mechanisms. This synergism enhanced the STING cascade through releasing DNA fragments into the cytosol for detection by cGAS within immune cells of the TME (62). Mice bearing 4T1 (breast cancer) tumours following intravenous treatment with these manganese complexes, displayed an increase in mature dendritic cells of 45% in lymph nodes versus control treatments (95), while enhancing the production of pro-inflammatory cytokines and type I interferons, resulting in a boost in immunosurveillance and tumour resection (95).

Though their pre-clinical benefit in murine tumour models has been evident, metal-based complexes as direct/indirect STING agonists have been limited due to concerns regarding systemic adverse effects (96). Namely, platinum-based complexes have been found to exhibit poor selectivity affecting healthy cells and resulting in haematologic toxicity and myelosuppression among others in phase I/II clinical trials for high-grade gliomas (NCT01644955, n=10, single-arm; NCT04851834, n=12, four-arm) (97, 98). There is also an ongoing concern regarding the potential of development secondary immune-related cancers following metal-based drug therapy, thus limiting their overall therapeutic applications as indirectly acting STING agonists, despite their interaction with the pathway (97). **Table 2** summarises all the agonists that have been mentioned, including their development stage and any clinical data available.

**Table 2:** A summary of all STING agonists in clinical trials.

STING agonist	Class	Stage	Administration	Participants	Clinical Ref	Outcomes
ADU-S100	CDN	Clinical (phase II, terminated)	Intratumoural	47 106 16	NCT02675439, NCT03172936, NCT03937141	No substantial anti-tumour activity observed/sponsor decision
MK-1454	CDN	Clinical (phase I/II, completed)	Intratumoural	156 8	NCT03010176 NCT04220866	CXCL10, IFNγ and IL-6 elevated at 2-4hrs and peak at 6 to 8hrs, plateau at 24hrs 50% of phase II participants with combination pembrolizumab had a complete or partial tumour regression
TAK-676	CDN	Clinical (phase II, active)	Intravenous infusion	248	NCT04420884	N/A
DMXAA	Small molecule	Clinical (phase III, terminated)	N/A	1285	NCT00662597	N/A
ISM5939	ENPP1 inhibitor	Clinical (phase I, active)	Oral	25	NCT06063681	N/A
2’3’-cGAMP (ONM-501)	CDN	Clinical (phase I, active)	Intratumoural	168	NCT06022029	N/A

## Challenges and limitations of STING agonism in brain cancers

Despite the mixed results of STING agonists in clinical trials, STING activation remains an enticing target for solid tumours including gliomas, with recent research focusing on improving their bioavailability and stability. Particularly, with a focus on overcoming the limited permeation of CDN-based STING agonists which are negatively charged and hydrophilic, through the cell membrane of target cells, antigen-presenting cells (99–101). As demonstrated by the clinical trials involving STING agonists in solid tumours, clinical data available explicitly for brain tumours has been limited. Even though we provide an outlook on STING agonists from existing data on solid tumours, this highlights a critical knowledge gap requiring further investigation to implement these agents clinically for that indication.

**Figure 4:**
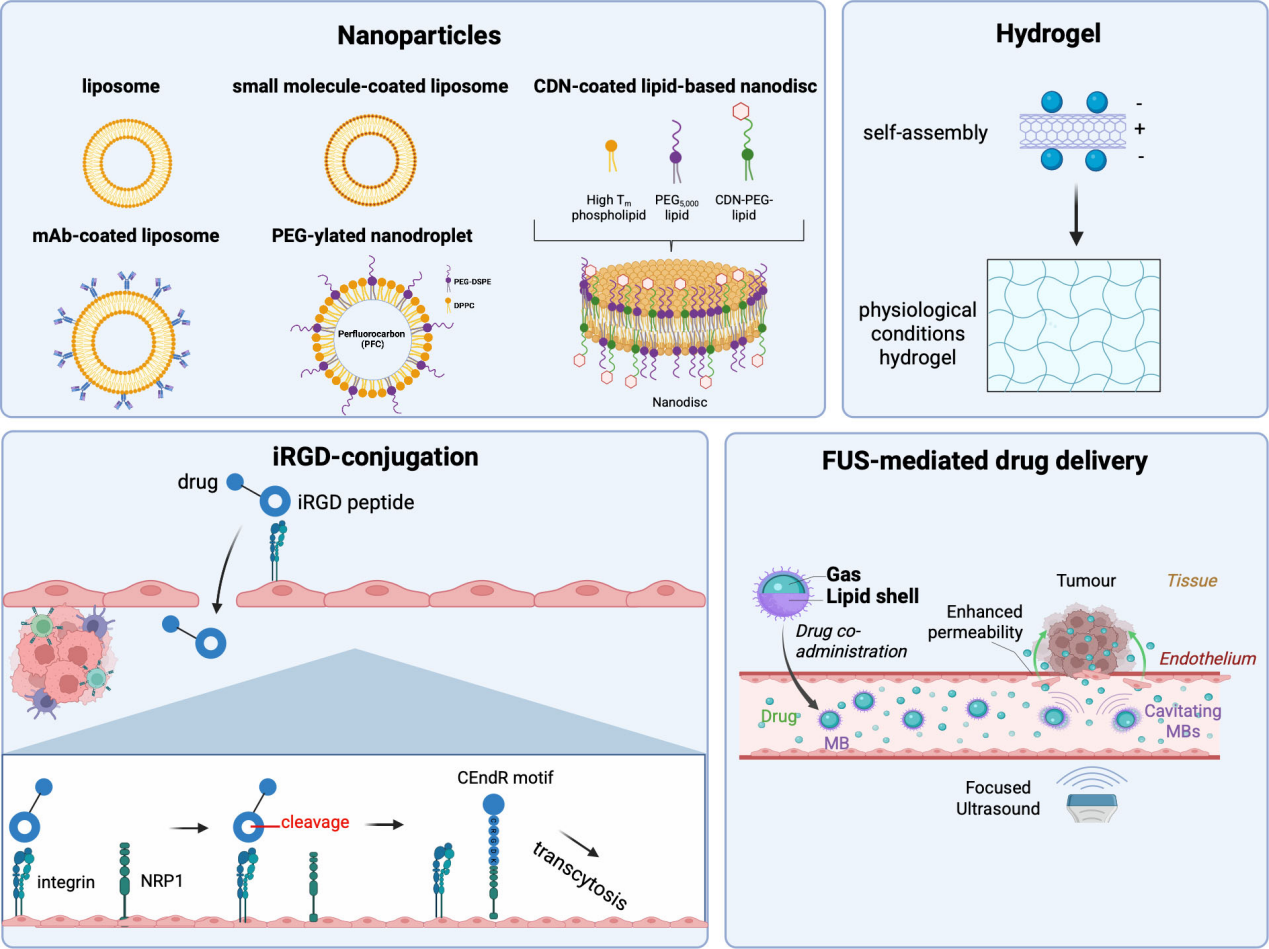
Strategies of enhancing STING agonist delivery. Schematic showing the different avenues available to overcome drug delivery limitations with STING agonists. These range from nanoparticles, self-assembling hydrogels and microbubbles in conjunction with focused ultrasound for increased bioavailability to tumours. The nanoparticles most often used for this indication are liposome-based. They can act as drug delivery systems which can shield CDN-based STING agonists from phosphodiesterase enzymes and thus resist degradation systemically through polyethylene glycol (PEG) on the lipid shell. They can also facilitate combination with other immunotherapies such as monoclonal antibodies, through coating of the nanoparticle lipid surface. More recently, nanodroplets with a perfluorocarbon core and liposome shell have been investigated for this. These drug delivery modalities can be conjugated to an iRGD peptide to facilitate increased update and endocytosis by the tumour dependent on integrin expression. A self-assembling hydrogel has been developed to facilitate the sustained release of CDNs when administered intratumourally in regions limited for drug delivery, such as the brain. Focused ultrasound-mediated drug delivery in conjunction with microbubbles has increased in popularity due to its ability to permeate biological barriers such as endothelial linings from vessels to access tumour sites from the circulation. Figure created in Biorender.com.

It is worth noting that proteins of the cGAS/STING pathway are expressed in most host tissues and thus future therapeutic approaches aimed at STING agonist drug delivery should avoid global host immune system activation. Systemic activation could result in debilitating adverse effects such as a cytokine storm (24). This excessive release of cytokines accompanied by inflammatory cell death, appeared to depend on the signal strength of STING activation with higher doses of STING agonist di-ABZI inducing healthy monocyte apoptosis (102). Further research aiming at the systemic delivery of STING agonists should determine optimal dosing regimens that produce an anti-tumour response while mitigating these effects. This risk can be mediated through additional research for targeted drug delivery systems to minimise off-target adverse effects like apoptotic cell death of healthy cells. Moreover, the specific threshold of 2’3’-cGAMP molecules necessary to trigger the STING downstream signalling cascade for antitumour effects remains elusive for brain malignancies (24). Several research groups have explored various means of boosting the efficacy of STING agonists for brain cancer treatment (**Figure 4**).

Finally, all STING agonists that have just been mentioned have been highlighted to have poor membrane permeability, which contributes to their poor permeation of the blood-brain-barrier for application in brain tumours. Permeability coefficients for these agents have also not yet been reported, highlighting a knowledge gap that would contribute to the collective understanding and implementation of these agents clinically for brain malignancies.

## Nanoparticles as a potential drug delivery system

Overcoming STING agonist delivery limitations has been of interest to the scientific community, with nanoparticle-based drug delivery systems being at the forefront. Zhang et al. were able to encapsulate STING agonists in bridging-lipid nanoparticles, while coating them in anti-PDL-1 antibodies, mediating robust antitumour efficacy in an orthotopic CT-2A murine model of glioblastoma, eradicating the engrafted tumour in 70% of tumour-bearing mice (103). These drug-loaded lipid nanoparticles induced glioma-associated phagocytosis by myeloid cells of the TME while inducing a T cell-supportive myeloid cell phenotype (103). Another group also developed a liposome for the delivery of the nucleotide-based STING agonist cyclic di-GMP, maximising the delivery of the molecule to activate the associated cGAS/STING pathway in antigen-presenting cells in mouse tumour models (104). Specifically for solid tumours, the inhalation of phosphatidylserine-coated liposomes loaded with CDN 2’3’-cGAMP in murine models of lung metastases was found to distribute the molecule to the lungs and stimulate the cGAS/STING pathway in antigen-presenting cells – specifically a 400-fold increase in IFNβ1 and a 200-fold increase in IFNα1 expression were observed in bone-marrow derived macrophages (105).

Liposomes can be formulated for increased stability in the blood circulation using cholesterol and pegylated lipids in optimal ratios to accommodate 2’3’-cGAMP or cyclic di-GMP (106). Atukorale et al. (107), have formulated an immunostimulatory liposome for the co-delivery of cyclic di-GMP and monophosphorylate lipid A, a toll-like receptor 4 (TLR4) agonist, for the treatment of pancreatic ductal adenocarcinoma, an advanced solid tumour (107). They subsequently found an 11-fold increase in the levels of IFN-β, improving immunosurveillance in the TME *in vivo*.

As previously mentioned, the negative charge of CDN-based STING agonists such as 2’3’-cGAMP hinders their internalisation into the cytosol of cells of interest. Cationic lipids such as 1,2-dioleoyl-3-trimethyl-ammonium-propane (DOTAP) have been implicated in the fabrication of nanoparticles to overcome this (99). DOTAP-based liposomes have been used in the encapsulation of ADU-S100 which promoted the maturation of dendritic cells, through upregulation of CD40, CD80 and CD86 on the surface of bone-marrow derived dendritic cells following 6hr incubation with the liposome (108). DOTAP-based liposomes demonstrated an encapsulation efficiency of ADU-S100 of ~100%. This was accompanied by a 200,000 pg/ml increase in cytokines CXCL10 and a 7000pg/ml increase in CCL5, both of which are cytokines associated with STING pathway activation, 16h post intravenous administration, in orthotopic MC38 tumour-bearing C57BL/6 mice (99).

The intratumoural injection of the liposomes significantly improved the survival rates of the mice, with 35% recovering completely from the primary tumour indicating the intratumoural availability of these agents is the main challenge in STING therapy (109). Dane et al. investigated other nanoformulations with the ability to accommodate STING agonists such as through their conjugation to lipids to form amphiphilic molecular assemblies, such as nanodiscs (110). The amphiphiles can then be utilised to prepare nanoparticles through nanoprecipitation, improving the overall encapsulation efficiency of STING agonists. Intravenous injection of the resulting liposome-CDN nanoparticles effectively reduced tumour growth in mouse models of colon cancer (110).

To overcome limitations regarding the off-target inflammation which can be observed with the systemic administration of STING agonists like cGAMP, targeting folate receptors have been suggested. Folate receptors are highly expressed on the surface of most tumour cells while being of low expression in healthy cells (111). This can facilitate tumour cell uptake due to receptor mediated endocytosis. Dane at al., have utilised cGAMP-encapsulated liposomes to modify folate on the surface, rendering liposomes able to target cancer cells that overexpress these receptors (112). Folate acid-mediated TME targeting liposomes were found to activate the STING-IRF3 pathway in tumour-associated antigen-presenting cells and induce type I interferon secretion. They demonstrated tumour regression and complete responses in 75% of MC38 (colon adenocarcinoma) tumour-bearing mice following a single dose of 100nmol of ADUS-100 embedded in the nanodisc (110). A cyclic peptide composed of 9 amino acids including an Arg-Gly-Asp (RGD) motif named iRGD, possesses a high binding affinity for integrins which are abundant in tumour vasculatures. Following integrin binding, iRGD is proteolytically cleaved to expose the cryptic CendR motif, which in turn binds to NRP-1, triggering an active endocytic step to aid in the internalisation of iRGD, and thus of the therapeutic entity it is conjugated to. It is worth noting that integrin α_v_β_3_ has been found to be overexpressed in glioma tumours, highlighting the potential of iRGD-conjugated drug delivery systems such as nanoparticles for anti-glioma therapeutic applications (**Figure 4**) (113). The efficacy of iRGD-conjugated nanoparticles however is still to be determined in the context of high-grade brain tumours.

Within the field of nanoparticles, spherical nucleic acids (SNAs; nucleic acids coupled on to gold nanoparticles) have emerged as promising nucleic acid carrier as cancer therapeutics. A report on the development of cGAS-activating SNAs that consist of gold nanoparticle cores functionalised with a shell of densely packed interferon-stimulatory DNA oligonucleotides, shows that the SNAs induced proinflammatory response and when administered with immune checkpoint inhibitors they abolished glioblastoma tumours in mice (114).

Overall, the nanoparticle approach is a relatively rapid, low-cost and well-established method for drug encapsulation, which can be applied to accommodate a variety of STING agonist types, while demonstrating anti-tumour efficacy in animal models. The observations made by different groups render nanoparticles as a therapeutic route which offers significant promise for the application of these types of nanoparticles to other types of solid tumour such as high-grade gliomas in the future, bypassing the limitations of the free drug alternatives for STING.

## Hydrogels as STING agonists sustained delivery systems

Hydrogels are three-dimensional cross-linking networks consisting of hydrophilic polymers able to establish a mesh-like network to preserve their structural integrity while being malleable (115, 116). Currently, there are more than 30 hydrogels which are FDA-approved for clinical applications including commercially successful INFUSE (Medtronic, Inc) and Vantas (Endo Pharmaceuticals) (99). Since most STING agonists being investigated in the clinic rely on intratumoural injections for their administration, hydrogels offer great promise for the delivery of STING agonists through the formation of scaffolds for the slow release of drug molecules. Molecules such as hyaluronic acid have been used for the development of 3D scaffolds loaded with immunotherapy and resulted in the slow release of the drug while significantly reducing the occurrence of lung metastases in murine models of breast cancer (117). Negatively charged CDNs such as cyclic deadenylate monophosphate (CDA) can become embedded within the hydrogel due to their highly negative charge within the physiological conditions of the TME. Indeed, intratumoural injection of a self-assembling camptoth-ecin-based hydrogel with this type of CDNs, was found to elicit tumour regression, demonstrating a surprising 100% survival rate in a subcutaneous GL-261 murine model of glioblastoma (118). The local injection of this hydrogel elicited an infiltration of lymphocytes, macrophages and natural killer (NK) cells within the TME, demonstrating the tumour availability is necessary for efficacy. Hydrogels may initially release the STING agonists rapidly which can result in excessive initial activation of the innate immune response and an excessive systemic inflammatory response, contributing to off-target effects (99). Yet, their efficiency in the brain as an implant releasing drugs after brain tumour resection is to be investigated. When it comes to applications of the hydrogels for deep seeded inoperable brain tumours such as DMGs, the complex anatomical location of these tumours constitutes a significant limitation as they would need to be injected to the area, for example the pons in the case of DMG. Nonetheless, hydrogels do possess low toxicity and good biocompatibility with a high capacity for drug loading in the case of CDNs for STING pathway activation (119, 120).

## Microbubbles and focused ultrasound as a means for overcoming the blood-brain-barrier

Another promising therapeutic avenue to maximise drug delivery of these agents would be the use of microbubble-assisted focused ultrasound (FUS) for targeted delivery of therapeutic entities into the brain parenchyma. FUS utilises low-frequency ultrasound waves to stimulate controllable cavitation of systemically injected microbubbles (121, 122). Microbubbles are gas-filled emulsions with a gaseous core stabilised by a surrounding shell consisting of lipids and other biocompatible molecules (123). Cavitation entails the volumetric oscillations triggered by the FUS field, characterised by alternating phases of contraction and expansion. The cavitation process generates sufficient mechanical energy in the form of shear stress, enabling the opening of tight junctions of cell layers within the blood-brain barrier (BBB) (124). The size of microbubbles allows for the loading of drugs of interest through conjugation for targeted drug delivery (125). Through cavitation-induced permeation of the BBB, the drugs of interest can permeate through the endothelial cells layer and allow for more targeted delivery of the drug while minimising invasiveness in complex anatomical areas such as the brain.

Many clinical trials have focused on novel means of encapsulating anti-cancer agents for increased stability in the circulation involving nanoparticles. In the context of activation of the cGAS/STING pathway, Li et al., found that ultrasound-responsive microbubbles can be used for sonoporation in tumour cells to enable the entry of labile nucleic acids such as CDNs into the cytosol, to activate the downstream STING pathway in antigen-presenting cells *in vitro* (121). Remarkably, this method demonstrated significant therapeutic efficacy in murine tumour models of solid cancers with tumour growth inhibition and extended rates of survival in metastatic breast cancer models (126). These findings highlight the potential of utilising novel nanoparticle-based drug delivery systems in conjunction with FUS for the delivery of STING agonists to the cytosol of cells of interest, bypassing their limited stability as free drugs in the systemic circulation, and improving their bioavailability to the brain. FUS has also been extensively investigated for high-grade glioma therapy in pre-clinical models and clinical trials, having an established overall safety profile (127–132). Studies using these methods showed increased antitumour efficacy and prolonged survival in murine glioma models (133, 134). The clinical trials involving FUS for the treatment of high-grade gliomas have been reviewed elsewhere (123, 135).

It is hence evident that the future of STING agonist delivery for high-grade gliomas while boosting anti-tumour responses, lies within the intersection of nanoparticle encapsulation to boost stability, while bypassing physical barriers to boost the bioavailability of the drug to the area of interest. This highlights an interesting research area which shows significant promise, though requiring further research to determine the dosage and efficacy of small molecule and newer generation STING agonists when encapsulated by nanoparticles, ensuring compatibility with FUS.

The different delivery strategies mentioned to boost bioavailability of STING agonists to the brain, have been highlighted in **Table 3**.

**Table 3:** Strategies to overcome STING agonist delivery limitations.

Delivery method proposed	Advantages	Disadvantages	Suitability for brain tumours	Clinical trials and/or important findings
Nanoparticles including iRGD targeting	Overcoming BBB size limitations Systemic administration Limited off-target toxicity iRGD conjugation highly targeted for high-grade gliomas Permeation of tumour vasculature	Optimal dosing and formulations ought to be optimised For iRGD targeting, α_v_β_3_ and α_v_β_5_ integrin expression needs to be validated first	Yes	DOTAP-based liposomes [1] TLR4 and cyclic di-GMP co-delivery liposomes [2] Anti-PDL-1 Ab-coated lipid nanoparticles [3] iRGD-coated nanoparticles [4] Spherical nucleic acids [5]
Hydrogels	Scaffolds for slow release of STING agonist over time Compatible with negatively charged CDNs Low toxicity	Implantation difficulty	Yes	CDA-hydrogel in glioblastoma [6]
Microbubbles and Focused Ultrasound	Non-invasive transient BBB permeation Clinically proven as safe Investigated in high-grade glioma	Possible microhaemorrhages Parameterisation of ultrasound required	Yes	Targeting APCs using FUS for sonoporation and 2’3’-cGAMP delivery [7] iRGD-modified liposomes phase transition to microbubbles in a 4T1 tumour model with FUS and increase NK cell populations in the TME [8]

1. Koshy, S.T., et al., Liposomal Delivery Enhances Immune Activation by STING Agonists for Cancer Immunotherapy. Adv Biosyst, 2017. 1(1-2).

2. Kane, G.I., et al., Super-adjuvant nanoparticles for platform cancer vaccination. Cell Reports Medicine, 2025. 6(10).

3. Zhang, P., et al., STING agonist-loaded, CD47/PD-L1-targeting nanoparticles potentiate antitumor immunity and radiotherapy for glioblastoma. Nature Communications, 2023. 14(1): p. 1610.

4. Zhou, Q., et al., Nanoparticle-Mediated STING Agonist Delivery for Enhanced Cancer Immunotherapy. Macromol Biosci, 2021. 21(8): p. e2100133.

5. Mahajan, A.S., et al., cGAS-agonistic spherical nucleic acids reprogram the glioblastoma immune microenvironment and promote antitumor immunity. Proceedings of the National Academy of Sciences, 2025. 122(45): p. e2409557122.

6. Wang, F., et al., Tumour sensitization via the extended intratumoural release of a STING agonist and camptothecin from a self-assembled hydrogel. Nat Biomed Eng, 2020. 4(11): p. 1090-1101.

7. Li, X., et al., Cancer immunotherapy based on image-guided STING activation by nucleotide nanocomplex-decorated ultrasound microbubbles. Nature Nanotechnology, 2022. 17(8): p. 891-899.

8. Hu, C., et al., Low-Intensity Focused Ultrasound-Responsive Phase-Transitional Liposomes Loaded with STING Agonist Enhances Immune Activation for Breast Cancer Immunotherapy. Cancers (Basel), 2024. 16(21).

## Combination of therapeutic strategies

An interesting topic of discussion has also been the investigation into combination therapies with STING agonism for malignancies. Indeed, combinatorial anti-cancer effects have been demonstrated with PARP inhibitors. Double-stranded DNA-induced activation of the cGAS/STING pathway in myeloid cells has been found to be dependent on PARP inhibition and enhances the efficacy of PARP inhibitors altogether. This effect has been demonstrated in pre-clinical breast and ovarian cancer models whereby treatment with PARP inhibitor Olaparib resulted in the release of ds-DNA fragments by tumour cells, in turn activating the downstream STING cascade intratumourally in dendritic cells, stimulating the downstream type I interferon response and T cell recruitment (38, 136). This combinatorial effect however is yet to be explored in the context of brain malignancies.

Interestingly, there has also been evidence of crosstalk between STING agonists and T cell receptor (TCR) T cells *in vivo*, whereby TCR-like CAR T cells pre-treated with STING agonist di-ABZI, significantly suppressed tumour growth in melanoma tumour-bearing mouse models (137). The CD47 receptor is highly expressed in high-grade gliomas and associated with poorer clinical outcomes. CD47 inhibition has previously been found to enhance the efficacy of STING-targeting therapy via activating phagocytic cells in a model of solid tumours, highlighting the potential of combination therapy of STING agonism and CD47 antagonism (138, 139). Combination therapies including checkpoint inhibition on tumour cells via PD-L1 blockade and CD47 in conjunction with STING agonist delivery in lipid nanoparticles demonstrated enhanced phagocytic ability and antigen presentation of myeloid lineage immune cells. Interestingly, neither free drug combinations were able to induce T cell infiltration and activation with the same potency as the drug-loaded nanoparticles, nor were they able to eradicate the tumour from glioma-bearing mice; overall highlighting the importance of a multi-drug-delivery system for the manipulation of the immunosuppressed TME in these tumour types. The role of other myeloid populations such as microglia have also been suggested to be reprogrammed in the context of diffuse high-grade gliomas using drug-loaded nanoparticles (103, 140).

The potential for combination therapy including immune checkpoint inhibitors with STING agonists has largely emerged from their ability to boost immunosurveillance in the TME. STING agonists are thought to prime the innate immune response through the release of type I IFNs, which in turn result in the activation of antigen-presenting cells such as dendritic cells and macrophages. Following chemokine secretion, namely CXCL9/10, T cell infiltration increases, in turn resulting in the tumour becoming more immunogenic. Immune checkpoint inhibitors can hence block the inhibitory signals through PD-1/PD-L1 and CTLA-4 blockade, allowing for the cytotoxic T cell responses to occur (20). Indeed, this rationale has been validated in preclinical models, whereby glioma models demonstrate that STING agonism can rescue response in immune checkpoint-resistant tumours (17). A potential triple therapy with radiotherapy is also currently being explored in high-grade gliomas, due to the release of additional DNA fragments that in turn act as cyclic dinucleotides and thus, result in STING protein binding and activation (51). Though the potential of the aforementioned combinatorial therapies is immense for the treatment of brain tumours, the *in vivo* data remains limited, with clinical studies currently unavailable.

## Conclusion

The exploration of STING agonism as a therapeutic avenue for high-grade gliomas and other CNS malignancies is a promising yet complex frontier. The STING pathway plays a pivotal role in mediating innate and adaptive immune responses through the production of type I interferons and pro-inflammatory cytokines, offering a robust anti-tumour mechanism in the immunosuppressive microenvironment of gliomas. However, the translation of these findings into clinical practice remains hindered by challenges such as poor drug stability, limited bioavailability, and the need for precise targeting to avoid off-target immune activation.

Emerging technologies such as nanoparticle-based drug delivery systems, liposomal encapsulation, hydrogels, and FUS are paving the way for more effective and targeted delivery of STING agonists. These advancements are crucial for overcoming physical barriers, such as the blood-brain barrier, and ensuring the stability and bioactivity of therapeutic agents in the systemic circulation. Depending on future findings regarding the epigenetic silencing of the STING pathway in the brain parenchyma and neoplastic cells, epigenetic targeting could constitute an opportunity to de-repress STING, enabling the recognition of cytosolic ds-DNA within cancer cells. Overall, the aim should be to boost immunosurveillance in the TME via myeloid cell-independent avenues.

While promising pre-clinical data and early clinical trials have highlighted the therapeutic potential of STING agonism, further research is needed to optimise delivery methods, determine appropriate dosing regimens, and better understand the mechanistic nuances of STING activation in brain cancer. Particularly, addressing the delicate balance between immune activation and systemic toxicity will be critical for the successful clinical translation of these approaches.
